# The prognostic significance of right paratracheal lymph node dissection numbers in right upper lobe non-small cell lung cancer

**DOI:** 10.1007/s13304-024-01778-7

**Published:** 2024-02-28

**Authors:** FengNian Zhuang, JunPeng Lin, WeiJie Chen, XiaoFeng Chen, YuJie Chen, PeiYuan Wang, Feng Wang, ShuoYan Liu

**Affiliations:** 1 Department of Thoracic Oncology Surgery, Clinical Oncology School of Fujian Medical University, Fujian Cancer Hospital, No. 420 Fuma Road, Fuzhou, 350001 Fujian China; 2 Fujian Key Laboratory of Translational Cancer Medicine, Fuzhou, China; 3 Fujian Provincial Key Laboratory of Tumor Biotherapy, Fuzhou, China

**Keywords:** Non-small cell lung cancer, Lymph node dissection, Lymphadenectomy, Right upper lobe, Lymph node number

## Abstract

**Background:**

The number of dissected lymph nodes is closely related to the prognosis of patients with non-small cell lung cancer. This study explored the optimal number of right paratracheal lymph nodes dissected in right upper non-small cell lung cancer patients and its impact on prognosis.

**Methods:**

Patients who underwent radical surgery for right upper lobe cancer between 2012 and 2017 were retrospectively enrolled. The optimal number of right paratracheal lymph nodes and the relationship between the number of dissected right paratracheal lymph nodes and the prognosis of right upper non-small cell lung cancer were analysed.

**Results:**

A total of 241 patients were included. The optimal number of dissected right paratracheal lymph nodes was 6. The data were divided according to the number of dissected right paratracheal lymph nodes into groups RPLND + (≥ 6) and RPLND- (< 6). In the stage II and III patients, the 5-year overall survival rates were 39.0% and 48.2%, respectively (P = 0.033), and the 5-year recurrence-free survival rates were 32.8% and 41.8%, respectively (P = 0.043). Univariate and multivariate analyses revealed that among the stage II and III patients, ≥ 6 right paratracheal dissected lymph nodes was an independent prognostic factor for overall survival (HR = 0.53 95% CI 0.30–0.92 P = 0.025) and recurrence-free survival (HR = 1.94 95% CI 1.16–3.24 P = 0.011).

**Conclusions:**

Resection of 6 or more right paratracheal lymph nodes may be associated with an improved prognosis in patients with right upper non-small cell lung cancer, especially in patients with stage II or III disease.

## Background

The morbidity and mortality of lung cancer worldwide are high, and more than 80% of lung cancer cases are non-small cell lung cancer (NSCLC), which is the most common type of lung cancer [1].The current standard surgical approach for resectable NSCLC is lobectomy with mediastinal lymph node dissection. Mediastinal lymph node dissection is the key to surgery and is related to accurate staging and complete tumor resection. For mediastinal lymph node dissection in non-small cell lung cancer, the National Comprehensive Cancer Network (NCCN) guidelines recommend resecting at least three mediastinal lymph node stations, including Group 7, and there is no exact requirement for the specific scope of dissection [2]. There are differences in the prognosis of lung cancer in different lung lobes [3]. Lung cancer is most likely to occur in the right upper lobe, the incidence rate of which ranges from 25% to 40% [4, 5], and is more prone to invasive cancer [6]. However, the degree of lymph node drainage used for lung cancer differs among different lung lobes [3, 4], and the dissection of different mediastinal lymph node stations in the same lung lobe affects patient prognosis. Therefore, the extent of mediastinal lymph node dissection in different lung lobes cannot be generalized. Many studies have confirmed that right paratracheal lymph nodes are the specific drainage pathway for right upper lobe lung cancer, and dissection of the right paratracheal lymph nodes is also an independent prognostic factor for right upper lobe lung cancer [7, 8]. Therefore, right paratracheal lymph node dissection is highly important in the treatment of upper right lung cancer.

For many other types of cancer such as gastrointestinal cancer and breast cancer, the NCCN guidelines recommend the number of resected lymph nodes [9]. However, for lung cancer, there is no recommendation in the current guidelines. Many studies have shown that the number of lymph nodes dissected is an independent factor for survival in patients with non-small cell lung cancer, and the recommended number of lymph nodes dissected ranges from 10 to 20 [10–12]. However, few studies have focused on the number of lymph nodes that need to be dissected at different mediastinal stations. Therefore, it is worth investigating whether the number of mediastinal lymph nodes dissected at different stations affects the survival of patients with NSCLC. In this study, we evaluated the importance of survival in patients with right upper lobe non-small cell lung cancer according to the number of dissected right paratracheal lymph nodes.

## Methods

### Inclusion criteria and exclusion criteria

A retrospective analysis was performed on patients who underwent radical surgery for primary right upper lobe cancer at Fujian Cancer Hospital from 2012 to 2017. All patients underwent standard preoperative evaluation, including chest computed tomography (CT), abdominal color Doppler ultrasound, brain magnetic resonance imaging (MRI), bone emission computed tomography (ECT), or positron emission tomography-computed tomography (PET-CT), to rule out metastatic disease. All the surgeries were performed by thoracic surgeons with more than 5 years of surgical experience. All resected specimens were fixed with 10% formalin and embedded in paraffin. Serial 4-mm sections were stained with haematoxylin–eosin. Specimen evaluation was completed by more than two pathologists, and the results included the number of lymph nodes, internal structure, capsule integrity, and volume of the lymph nodes. Pathological staging was based on the eighth edition of the TNM staging criteria. According to the International Associations for the Study of Lung Cancer (IASLC) lymph node map, the numbers of right paratracheal lymph nodes are 2R (right upper paratracheal lymph nodes) and 4R (right lower paratracheal lymph nodes) [13].

The inclusion criteria were as follows: 1. All patients were older than 18 years and provided signed informed consent; 2. All patients underwent standard lobectomy or sleeve resection plus mediastinal lymph node dissection, either by open or minimally invasive methods; 3. Mediastinal lymph node dissection involved at least three stations, including groups 2R, 4R, and 7. Dissections of groups 10, 11, and 12 were also performed. Groups 13 and 14 were separated by lung lobe samples after the operation. 4. At least 6 lymph nodes were dissected, including 3 mediastinal lymph nodes, and the number of right paratracheal lymph nodes was at least 2.

The exclusion criteria were as follows: 1. Lung cancer: small cell lung cancer; 2. Metastatic cancer; 3. Neoadjuvant therapy (including chemotherapy, radiotherapy, targeted therapy, and immunotherapy); 4. Lymph node dissection did not meet the requirements of the NCCN guidelines; and 5. Sublobar resection was performed; 6. Incomplete clinical data; 7. Positive margins, non-R0 resection. Institutional ethics approval was obtained for the study.

### Postoperative follow-up

All patients were followed up after the operation. The follow-up methods included telephone follow-up and in-hospital medical record review. The specific methods used were to review patients every 3 months in the first year after surgery, every 5–6 months in the second and third years, and every 8–12 months in the fourth and fifth years. The review items included laboratory blood test results, chest CT, abdominal color Doppler ultrasound, brain MRI, bone ECT, and PET-CT if necessary.

The primary endpoint was overall survival (OS), assessed from the time of surgery to the time of death from any cause. The secondary endpoint was recurrence-free survival (RFS), which was assessed from the time of operation to the time of recurrence or metastasis of lung cancer.

### Statistical analysis

All the data were analysed using SPSS 26. The chi-square test was used for categorical variables, and the independent sample t test was used for continuous variables. Survival outcomes were analysed by Kaplan‒Meier analysis and tested by log-rank tests. The risk factors were analysed by Cox regression, and the significant variables among the single factors were analysed by multivariate analysis to evaluate the associations between each variable and OS and RFS. The hazard ratio (HR) and 95% confidence interval (CI) were calculated. P < 0.05 was considered to indicate statistical significance.

## Results

### Clinical and surgical data

After screening and exclusion criteria, a total of 241 patients were included in the study. The mean number of dissected mediastinal lymph nodes was 11.22 ± 5.64, and the mean number of dissected right paratracheal lymph nodes was 5.90 ± 3.49. The details are shown in Tables 1 and 2.

**Table 1:** Clinical data in all patients、Group RPLND + and Group RPLND-

Variables	Total (n = 241)	RPLND + (n = 118)	RPLND- (n = 123)	P value
Age(year)				0.099
Mean ± SD	59.57 ± 8.74	60.52 ± 8.32	58.66 ± 9.07	
Sex				0.063
Male	147(61.0)	79(66.9)	68(55.3)	
Female	94(39.0)	39(33.1)	55(44.7)	
Smoking history				0.28
Yes	114(47.3)	60(50.8)	54(43.9)	
No	127(52.7)	58(49.2)	69(56.1)	
GGO				0.148
Yes	48(19.9)	19(16.1)	29(23.6)	
No	193(80.1)	99(83.9)	94(76.4)	
Tumor location				0.63
Peripheral	183(75.9)	88(74.6)	95(77.2)	
Central	58(24.1)	30(25.4)	28(22.8)	
Tumor size(cm)				0.434
Mean ± SD	3.06 ± 1.71	3.15 ± 1.73	2.98 ± 1.69	
Pathological pattern				0.459
Adenocacinoma	174(72.2)	83(70.3)	91(74.0)	
Squamous	48(19.9)	24(20.3)	24(19.5)	
Adenosquamous	6(2.5)	4(3.4)	2(1.6)	
Others	13(5.4)	7(6.0)	6(4.9)	
Pleural invasion				0.817
Yes	82(34.0)	41(34.7)	41(33.3)	
No	159(66.0)	77(65.3)	82(66.7)	
Clinical T stage				0.806
1	125(51.9)	58(49.2)	67(54.5)	
2	89(36.9)	46(39.0)	43(35.0)	
3	18(7.5)	10(8.5)	8(6.5)	
4	9(3.7)	4(3.3)	5(4.0)	
Clinical N stage				0.820
0	170(70.5)	83(70.3)	87(70.7)	
1	29(12.0)	13(11.0)	16(13.0)	
2	42(17.4)	22(18.6)	20(16.3)	

SD Standard deviation, GGO Ground glass opacity

**Table 2:** Surgical data in all patients、Group RPLND + and Group RPLND-

Variables	Total	RPLND +	RPLND-	P value
	(n = 241)	(n = 118)	(n = 123)	
Minimally invasive				0.289
Yes	173(71.8)	81(68.6)	92(74.8)	
No	68(28.2)	37(31.4)	31(25.2)	
Surgical method				0.319
Lobectomy	230(95.4)	111(94.1)	119(96.7)	
Sleeve lobectomy	11(4.6)	7(5.9)	4(3.3)	
Operation duration(min)				0.572
Mean ± SD	151.16 ± 33.43	149.92 ± 31.91	152.36 ± 34.92	
Blood loss(ml)				0.158
Mean ± SD	166.22 ± 53.33	171.19 ± 57.17	161.46 ± 49.12	
Number of MLNs				< 0.05
Mean ± SD	11.22 ± 5.64	14.19 ± 4.880	8.37 ± 4.803	
Number of Station 7 LNs				0.542
Mean ± SD	3.59 ± 2.95	3.71 ± 2.999	3.48 ± 2.909	
Number of Station 10 LNs				0.183
Mean ± SD	3.62 ± 2.97	3.88 ± 3.282	3.35 ± 2.591	
Complication				0.893
Yes	36(14.9)	18(15.3)	18(14.6)	
No	205(85.1)	100(84.7)	105(85.4)	
Pathological T stage				0.83
1	83(34.4)	39(33.1)	44(35.8)	
2	130(53.9)	67(56.8)	63(51.2)	
3	22(9.1)	9(7.6)	13(10.6)	
4	5(2.6)	3(2.5)	2(2.4)	
Pathological N stage				0.617
0	178(73.9)	86(72.9)	92(74.8)	
1	24(10.0)	10(8.5)	14(11.4)	
2	39(16.1)	22(18.6)	17(13.8)	
TNM stage				0.658
1	149(61.8)	72(61.0)	77(62.6)	
2	48(19.9)	22(18.6)	26(21.1)	
3	44(18.3)	24(20.4)	20(16.3)	

*MLNs* Mediastinal lymph nodes, *LNs* Lymph nodes, *SD* Standard deviation

### Analysis of the optimal number of right paratracheal lymph nodes

The number of dissected right paratracheal lymph nodes ranged from 3 to 12, and the 5-year survival rate, hazard ratio (HR), and P value were calculated for each group. As shown in Table 3, when the number of dissected right paratracheal lymph nodes was 6, the highest 5-year survival rate (72%) was obtained when the number of dissected lymph nodes was ≥ 6, and the hazard ratio (HR) reached 0.657, which was the lowest among all the groups. The cut-off value was set as 6, and the data were divided into the RPLND + group (the number of dissected right paratracheal lymph nodes was more than or equal to 6) and the RPLND- group (the number of dissected right paratracheal lymph nodes was less than 6); follow-up analysis was subsequently performed.

**Table 3:** The optimal number of dissected right paratracheal lymph nodes

Number of Right Paratracheal LNs	5-year OS	HR	P value
< 3vs ≥ 3	63.5%vs70.7%	0.675	0.154
< 4vs ≥ 4	65.2%vs70.3%	0.688	0.136
< 5vs ≥ 5	67.7%vs69.7%	0.797	0.333
< 6vs ≥ 6	65.9%vs72.0%	0.657	0.070
< 7vs ≥ 7	68.7%vs69.2%	0.810	0.378
< 8vs ≥ 8	68.4%vs70.1%	0.807	0.412
< 9vs ≥ 9	69.8%vs65.4%	1.027	0.921
< 10vs ≥ 10	69.7%vs65.0%	1.085	0.783
< 11vs ≥ 11	69.9%vs60.0%	1.327	0.405
< 12vs ≥ 12	70.0%vs55.6%	1.382	0.387

LNs Lymph nodes, OS Overall survival, HR The hazard ratio

As shown in Tables 1 and 2, there were 118 patients in the RPLND + group and 123 patients in the RPLND- group. Only the number of dissected mediastinal lymph nodes significantly differed between the two groups, and the number in the RPLND + group was significantly greater than the number in the RPLND- group (14.19 ± 4.880 vs. 8.37 ± 4.803, P < 0.05).

### Analysis of the relationship between the optimal number of dissected right paratracheal lymph nodes and survival

**Fig. 1:**
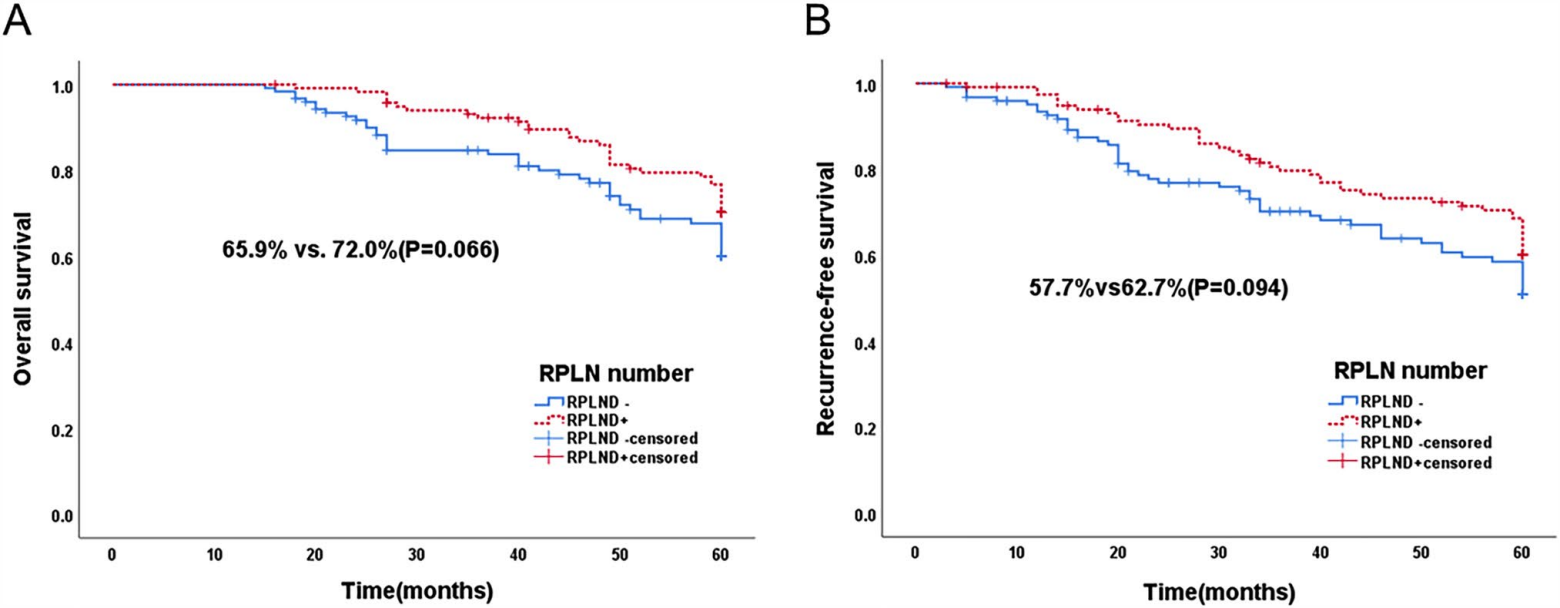
Overall Survival curves (**A**) and RFS curves (**B**) for all patients according to the number of lymph node dissections

The mean follow-up times in the two groups were 50.7 months and 50.6 months, respectively. As shown in Fig. 1, for the 5-year OS of all populations, the two sets of imaging data nearly overlapped before 16 months. After 16 months, the images of the two groups began to show differences, and the RPLND + group began to outperform the RPLND- group. The 5-year OS rates of the two groups were 65.9% and 72.0%, respectively (P = 0.066). At the 5-year RFS, the two groups of images began to diverge earlier, and the difference gradually increased. The difference reached a maximum at approximately 20 months. The 5-year RFS rates in the two groups were 57.7% and 62.7%, respectively (P = 0.094).

### Subgroup analysis

**Fig. 2:**
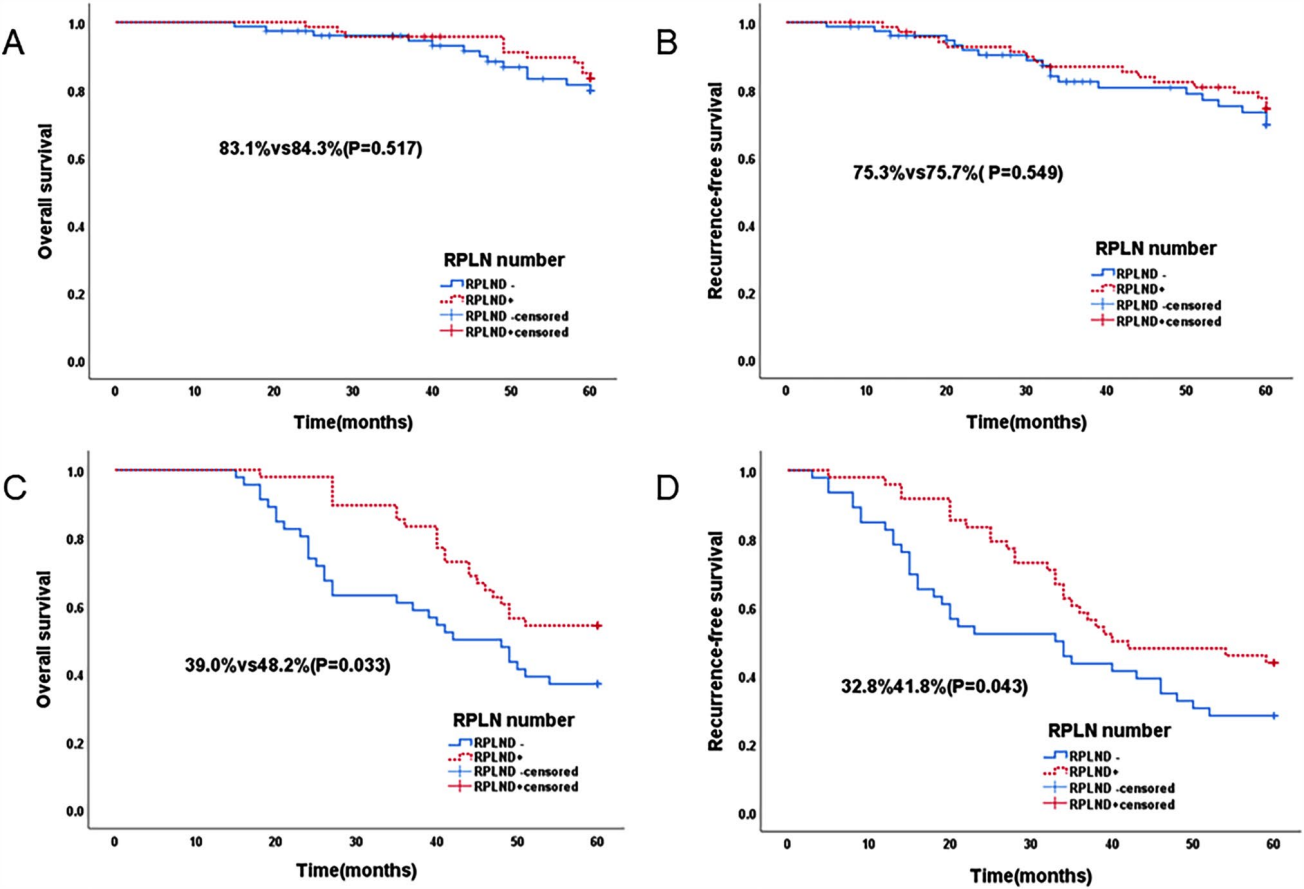
Overall Survival curves (**A**) and RFS curves (**B**) for Stage I patients and OS curves (**C**) and RFS curves (**D**) for Stage II and III patients according to the number of lymph node dissections

According to the stratified analysis according to tumor stage, in the stage I population, as shown in Fig. 2, the OS images of the two groups almost overlapped, and the difference began to appear after 40 months. The RPLND + group was slightly better than the RPLND- group, and the 5-year OS rates were 83.1% and 84.3%, respectively (P = 0.517). In RFS, the images of the two groups nearly overlapped, and there was a slight difference at 32 months; however, they tended to overlap again at approximately 48 months. The 5-year RFS rates of the two groups were 75.3% and 75.7%, respectively (P = 0.549).

In the stage II and III populations, as shown in Fig. 2, the OS images of the two groups showed obvious differences. Starting at approximately 14 months, the curves of the two groups opened and reached the maximum difference at 26 months. The 5-year OS rates of the two groups were 39.0% and 48.2%, respectively (P = 0.033). The difference between the two groups appeared earlier in RFS. The difference gradually increased over time. At 40 months, the difference gradually narrowed, and the tumors subsequently expanded until 60 months. The 5-year RFS rates were 32.8% and 41.8%, respectively (P = 0.043).

According to the above results, only patients in the II and III cohorts exhibited significant differences in survival, so the risk factors were analysed. According to the analysis of OS risk factors for patients in the Stages II and III cohorts, as shown in Table 4, N stage, tumor stage, and the number of dissected right paratracheal LNs ≥ 6 were risk factors according to univariate analysis. Multivariate analysis revealed that only ≥ 6 dissected right paratracheal LNs was an independent prognostic factor for OS (HR = 0.53 95% CI 0.30–0.92, P = 0.025). According to the analysis of risk factors for RFS, as shown in Table 5, the univariate analysis revealed that pleural invasion, postoperative adjuvant therapy, and ≥ 6 dissected right paratracheal LNs were risk factors for RFS in patients in the Stages II and III cohorts. Multivariate analysis revealed that pleural invasion (HR = 1.94 95% CI 1.16–3.24, P = 0.011) and ≥ 6 dissected right paratracheal LNs (HR = 0.66 95% CI 0.40–1.11, P = 0.013) were independent prognostic factors for RFS.

**Table 4:** Univariate and multivariate analyses of overall survival for patients in Stage II and III

	Univariate analyses			Multivariate analyses		
	HR	95% CI	P	HR	95% CI	P
Age						
< 65	1					
≥ 65	0.97	0.53–1.77	0.911			
Sex						
Male	1					
Female	0.61	0.32–1.17	0.138			
Smoking history						
No	1					
Yes	1.14	0.66–1.99	0.638			
Pathological pattern						
Adenocacinoma	1					
Squamous	0.71	0.38–1.21	0.276			
Adenosquamous	0.92	0.13–6.70	0.931			
Others	0.64	0.20–2.08	0.454			
Pleural invasion						
No	1					
Yes	1.56	0.90–2.71	0.116			
T stage						
1	1					
2	0.91	0.42–2.00	0.818			
3	0.77	0.31–1.90	0.565			
4	1.1	0.29–4.14	0.891			
N stage						
0	1			1		
1	1.55	0.72–3.34	0.265	1.34	0.60–2.96	0.477
2	2.03	1.04–3.96	0.039	1.05	0.34–3.26	0.935
Stage						
2	1			1		
3	1.94	1.11–3.39	0.021	2.14	0.78–5.89	0.140
Adjuvant therapy						
No	1					
Yes	1.64	0.90–2.99	0.109			
Whether RPLND +						
No	1			1		
Yes	0.56	0.32–0.97	0.038	0.53	0.30–0.92	0.025

*HR* The hazard ratio, *CI* Confidence interval

**Table 5:** Univariate and multivariate analyses of Recurrence-free survival for patients in Stage II and III

	Univariate analyses			Multivariate analyses		
	HR	95% CI	P	HR	95% CI	P
Age						
< 65	1					
≥ 65	1.03	0.58–1.80	0.933			
Sex						
Male	1					
Female	0.75	0.42–1.33	0.322			
Smoking history						
No	1					
Yes	1.04	0.63–1.73	0.878			
Pathological pattern						
Adenocacinoma	1					
Squamous	1.01	0.59–1.75	0.969			
Adenosquamous	0.69	0.10–5.06	0.718			
Others	0.53	0.16–1.73	0.293			
Pleural invasion						
No	1			1		
Yes	1.82	1.09–3.03	0.022	1.94	1.16–3.24	0.011
T stage						
1	1					
2	0.79	0.38–1.66	0.793			
3	0.84	0.37–1.95	0.692			
4	1.86	0.57–6.06	0.304			
N stage						
0	1					
1	1.44	0.72–2.88	0.307			
2	1.82	0.99–3.34	0.052			
Stage						
2	1					
3	1.62	0.97–2.69	0.065			
Adjuvant therapy						
No	1					
Yes	2.13	1.21–3.74	0.048	2.07	1.16–3.67	0.120
Whether RPLND +						
No	1			1		
Yes	0.6	0.36–0.99	0.009	0.66	0.40–1.11	0.013

*HR* The hazard ratio, *CI* Confidence interval

## Discussion

Mediastinal lymph node dissection is an important step in lung surgery. In real-world clinical work, the scope and content of lymph node dissection are mostly based on the experience of the surgeon. Lung cancer that occurs in different lobes has its own lymphatic drainage pathways [3, 4], and mediastinal lymph node stations that need to be dissected in different lobes should be selected. Wang et al. showed that Group 4L LN dissection is an important prognostic factor for left-sided lung cancer [14]. Liu et al. showed that Group 3A LN metastasis is not uncommon in right-sided lung cancer, and is also an important prognostic factor [15].The 2R and 4R lymph nodes have the same anatomical chain and the same lymphatic drainage pathway, and there is no clear anatomical boundary between 2R and 4R [16]. A study by Naruke et al. showed that the upper mediastinal lymph nodes are the most common site of N2 metastasis in right upper lung cancer [17]. Cerfolio concluded that the 2R and 4R lymph nodes are the most common N2 metastatic sites of upper right lung cancer [18]. A retrospective analysis of 2070 patients revealed that 4R lymph nodes are the most common metastatic station for right lung cancer, especially for right upper lung cancer, and that 4R lymph node dissection is an important prognostic factor [19]. Rami-Porta suggested that complete resection of right upper lung cancer must include dissection of the upper and lower paratracheal segments (i.e., 2R, 4R) [20]. Therefore, right paratracheal lymph node dissection may impact the prognosis of upper right lung cancer patients. However, for upper right lung cancer, there are few relevant studies on the extent to which right paratracheal lymph nodes need to be dissected or whether reaching the optimal number of right paratracheal lymph nodes dissected affects the prognosis of upper right lung cancer patients. Our study revealed that the optimal number of dissected lymph nodes was 6, and when the number of right paratracheal lymph nodes dissected reached 6 or more, patients with upper right lung cancer could have a better prognosis.

An increasing number of studies have focused on the correlation between the number of lymph nodes dissected and the prognosis of lung cancer patients. A study by Nwogu included 25,887 patients who underwent lung cancer surgery; the results showed that the more mediastinal lymph nodes were dissected, the better the prognosis was, and the trend towards improvement was more obvious in patients with advanced tumor stages [21]. Wang et al. concluded that the number of lymph nodes dissected differed among different groups, which in turn led to differences in prognosis [22]. These research conclusions are also consistent with our research conclusions. In our study, there were more dissected lymph nodes in the RPLND + group than in the RPLND- group, and the outcome was better, especially for stage II and III patients. Similarly, the number of lymph node dissections at Station 7 was similar, and the difference in the number of mediastinal lymph nodes was mainly reflected in the difference in the number of right paratracheal lymph nodes. Therefore, the greater the number of right paratracheal lymph nodes dissected, the better the prognosis for patients with upper right lung cancer, especially for patients with advanced-stage disease. This approach can also remind thoracic surgeons to pay attention to right paratracheal lymph node dissection for patients with right upper lung cancer, especially for patients with advanced clinical stages. Most of the current studies focus on the total number of lymph nodes dissected. At present, few studies have focused on whether the difference in the number of right paratracheal lymph nodes dissected affects patient prognosis. Therefore, our research may be useful as a supplement.

In our study, although all patients underwent systematic lymph node dissection, the number of lymph nodes dissected still differed significantly. We believe that this is due to differences in the intensity of the dissections. The median number of lymph nodes in 99% of patients in the Z0030 study was found to be at least 6 LNs in at least 3 stations, and the median number of LNs that could be obtained at 4R stations was 4 [23]. Therefore, ≥ 6 right paratracheal lymph nodes acquisition is feasible. For stage I patients, whose risk of lymph node metastatic load is relatively low theoretically, the extent and intensity of lymph node dissection may not provide benefit, and the difference in the number of lymph nodes dissected may not affect the prognosis; these findings are also the same as the conclusions of some previous studies [7, 24].However, some studies have shown that the intensity of lymph node dissection should not be weakened even for stage I patients [25–27]. Therefore, the intensity of lymph node dissection in stage I NSCLC patients needs to be further explored.

According to the European Society of Thoracic Surgeons (ESTS) guidelines, the number of dissected lymph nodes, including hilar and mediastinal lymph nodes, is 6 [28]. However, there is no recommendation on the optimal number of dissected lymph nodes. The recommended number of lymph nodes dissected varies from study to study. Dai et al. analysed the optimal number of dissected lymph nodes for patients with different T stages and reported that the optimal numbers of dissected lymph nodes for patients with T1a, T1b, T1c, and T2a disease were 8, 9, 10, and 11, respectively [29]. Liang et al. included 38,806 patients in the Surveillance, Epidemiology, and End Results (SEER) database and 5,706 patients in the Chinese multicenter registration database. All the patients were stage I-IIIA lung cancer surgery patients, and it was concluded that 16 lymph nodes dissected could be used as the optimal number of dissected nodes [10]. Becker et al. also suggested that the optimal number of dissected lymph nodes for obtaining a better prognosis is 16 [30]. Many studies have recommended that the optimal number of lymph nodes dissected be between 10 and 20 [31–34]. These results are similar to those of our study, in which approximately 11 mediastinal lymph nodes were dissected. In addition, we found that the optimal number of right paratracheal lymph nodes dissected was 6 for upper right lung cancer. When the number of right paratracheal lymph nodes dissected reaches 6, disease recurrence can be delayed, and a better OS can be obtained, leading to a better prognosis. When analysing the reasons for the beneficial prognosis, we considered the following: ①. Right paratracheal lymph nodes are the specific drainage pathways for upper right lung cancer. Complete dissection can eliminate more potential tumor cells, especially for advanced tumor stages. ②. After complete dissection, the more potential tumors are detected, the more accurate the staging can be, and postoperative antitumor treatment can benefit patients more accurately.

This study has several limitations. First, this was a retrospective controlled study, and there are some unavoidable limitations. We look forward to additional prospective studies for further analysis. Second, this study included only the most frequently dissected lymph node station in the right upper lung for analysis; moreover, the analysis of lymph nodes in Groups 3, 8, and 9 was insufficient and needs to be further supplemented in follow-up studies.

## Conclusion

For right upper lung non-small cell lung cancer, the optimal number of right paratracheal lymph nodes dissected is 6. After reaching 6, recurrence-free survival and overall survival can improve, and a better prognosis can be obtained, especially for patients with stages II and III disease. Therefore, it is necessary to strengthen the quality control of right paratracheal lymph node dissection for patients with stage II and III right upper lung non-small cell lung cancer.

## Data Availability

The data repository is not available online.

## References

[1] Sung H et al (2021) Global cancer statistics 2020: GLOBOCAN estimates of incidence and mortality worldwide for 36 cancers in 185 countries. CA Cancer J Clin 71(3):209–249 33538338 10.3322/caac.21660

[2] Ettinger DS et al (2022) Non–small cell lung cancer, version 3.2022, NCCN clinical practice guidelines in oncology. J Nat Comprehensive Cancer Network 20(5):497–530 10.6004/jnccn.2022.0025 35545176

[3] Liang R-B et al (2018) Incidence and distribution of lobe-specific mediastinal lymph node metastasis in non-small cell lung cancer: data from 4511 resected cases. Ann Surg Oncol 25:3300–3307 30083835 10.1245/s10434-018-6394-9

[4] Cerfolio RJ, Bryant AS (2006) Distribution and likelihood of lymph node metastasis based on the lobar location of nonsmall-cell lung cancer. Ann Thorac Surg 81(6):1969–1973 16731115 10.1016/j.athoracsur.2005.12.067

[5] Asamura H et al (1999) Lobe-specific extent of systematic lymph node dissection for non–small cell lung carcinomas according to a retrospective study of metastasis and prognosis. J Thorac Cardiovasc Surg 117(6):1102–1111 10343258 10.1016/s0022-5223(99)70246-1

[6] Kinsey CM et al (2014) Invasive adenocarcinoma of the lung is associated with the upper lung regions. Lung Cancer 84(2):145–150 24598367 10.1016/j.lungcan.2014.02.002 PMC4004700

[7] Adachi H et al (2017) Lobe-specific lymph node dissection as a standard procedure in surgery for non–small cell lung cancer: a propensity score matching study. J Thorac Oncol 12(1):85–93 27553515 10.1016/j.jtho.2016.08.127

[8] Shapiro M et al (2013) Lobe-specific mediastinal nodal dissection is sufficient during lobectomy by video-assisted thoracic surgery or thoracotomy for early-stage lung cancer. Chest 144(5):1615–1621 23828253 10.1378/chest.12-3069

[9] Gradishar WJ et al (2020) Breast cancer, version 32020, NCCN clinical practice guidelines in oncology. J Nat Comprehensive Cancer Network 18(4):452–478 10.6004/jnccn.2020.0016 32259783

[10] Liang W et al (2017) Impact of examined lymph node count on precise staging and long-term survival of resected non–small-cell lung cancer: a population study of the US SEER database and a Chinese multi-institutional registry. J Clin Oncol 35(11):1162 28029318 10.1200/JCO.2016.67.5140 PMC5455598

[11] Saji H et al (2011) Prognostic impact of number of resected and involved lymph nodes at complete resection on survival in non-small cell lung cancer. J Thorac Oncol 6(11):1865–1871 21964529 10.1097/JTO.0b013e31822a35c3

[12] Osarogiagbon RU, Ogbata O, Yu X (2014) Number of lymph nodes associated with maximal reduction of long-term mortality risk in pathologic node-negative non–small cell lung cancer. Ann Thorac Surg 97(2):385–393 24266949 10.1016/j.athoracsur.2013.09.058 PMC3946669

[13] Rusch VW (2009) The IASLC lung cancer staging project a proposal for a new international lymph node map in the forthcoming seventh edition of the TNM classification for lung cancer. J Thoracic Oncol 4(5):568–577 10.1097/JTO.0b013e3181a0d82e 19357537

[14] Wang Y-N et al (2018) Clinical significance of 4L lymph node dissection in left lung cancer. J Clin Oncol 36(29):2935–2942 30148659 10.1200/JCO.2018.78.7101

[15] Liu C et al (2021) Clinical significance of station 3A lymph node dissection in patients with right-side non-small-cell lung cancer: a retrospective propensity-matched analysis. Ann Surg Oncol 28:194–202 32638165 10.1245/s10434-020-08786-y

[16] Riquet M et al (2014) Number of mediastinal lymph nodes in non-small cell lung cancer: a Gaussian curve, not a prognostic factor. Ann Thorac Surg 98(1):224–231 24820386 10.1016/j.athoracsur.2014.03.023

[17] Naruke T et al (1999) Lymph node sampling in lung cancer: how should it be done? Eur J Cardio-Thoracic Surg 16(Supplement_1):S17–S24 10.1016/s1010-7940(99)00178-5 10536940

[18] Cerfolio RJ, Bryant AS, Minnich DJ (2012) Complete thoracic mediastinal lymphadenectomy leads to a higher rate of pathologically proven N2 disease in patients with non-small cell lung cancer. Ann Thorac Surg 94(3):902–906 22776083 10.1016/j.athoracsur.2012.05.034

[19] Zhou D et al (2022) Prognostic significance of 4R lymph node dissection in patients with right primary non-small cell lung cancer. World J Surg Oncol 20(1):1–11 35778770 10.1186/s12957-022-02689-w PMC9248107

[20] Rami-Porta R et al (2005) Complete resection in lung cancer surgery: proposed definition. Lung Cancer 49(1):25–33 15949587 10.1016/j.lungcan.2005.01.001

[21] Nwogu CE et al (2012) Number of lymph nodes and metastatic lymph node ratio are associated with survival in lung cancer. Ann Thorac Surg 93(5):1614–1620 22440365 10.1016/j.athoracsur.2012.01.065 PMC5616176

[22] Wang W et al (2019) Impact of different types of lymphadenectomy combined with different extents of tumor resection on survival outcomes of Stage I non-small-cell lung cancer: a large-cohort real-world study. Front Oncol 9:642 31396479 10.3389/fonc.2019.00642 PMC6668052

[23] Darling GE et al (2011) Number of lymph nodes harvested from a mediastinal lymphadenectomy: results of the randomized, prospective American College of Surgeons Oncology Group Z0030 trial. Chest 139(5):1124–1129 20829340 10.1378/chest.10-0859 PMC3087457

[24] Hishida T et al (2016) Lobe-specific nodal dissection for clinical stage I and II NSCLC: Japanese multi-institutional retrospective study using a propensity score analysis. J Thorac Oncol 11(9):1529–1537 27249959 10.1016/j.jtho.2016.05.014

[25] Lardinois D et al (2005) Morbidity, survival, and site of recurrence after mediastinal lymph-node dissection versus systematic sampling after complete resection for non-small cell lung cancer. Ann Thorac Surg 80(1):268–275 15975380 10.1016/j.athoracsur.2005.02.005

[26] Tsai T-M et al (2022) Factors associated with nodal upstaging in clinical T1a-bN0M0 non-small cell lung cancers. Cancers 14(5):1277 35267588 10.3390/cancers14051277 PMC8909294

[27] Gabryel P et al (2023) Predictors of long-term survival of thoracoscopic lobectomy for stage ia non-small cell lung cancer: a large retrospective cohort study. Cancers 15(15):3877 37568693 10.3390/cancers15153877 PMC10416904

[28] De Leyn P et al (2014) Revised ESTS guidelines for preoperative mediastinal lymph node staging for non-small-cell lung cancer. Eur J Cardiothorac Surg 45(5):787–798 24578407 10.1093/ejcts/ezu028

[29] Dai J et al (2019) Optimal lymph node examination and adjuvant chemotherapy for stage I lung cancer. J Thorac Oncol 14(7):1277–1285 31009811 10.1016/j.jtho.2019.03.027

[30] Becker DJ et al (2018) Influence of extent of lymph node evaluation on survival for pathologically lymph node negative non–small cell lung cancer. Am J Clin Oncol 41(8):820–825 28301349 10.1097/COC.0000000000000379

[31] Ding H et al (2019) Survival and resected lymph node number during sublobar resection for N0 non-small cell lung cancer 2 cm or less. Ann Thorac Surg 107(6):1647–1655 30682353 10.1016/j.athoracsur.2018.12.024

[32] Krantz SB et al (2017) Improved lymph node staging in early-stage lung cancer in the national cancer database. Ann Thorac Surg 104(6):1805–1814 29102039 10.1016/j.athoracsur.2017.06.066

[33] Uimonen M et al (2023) Standard Lymphadenectomy for Esophageal and Lung Cancer: Variability in the Number of Examined Lymph Nodes Among Pathologists and Its Survival Implication. Ann Surg Oncol 30(3):1587–1595 36434484 10.1245/s10434-022-12826-0 PMC9908682

[34] Zhu Z et al (2021) A large real-world cohort study of examined lymph node standards for adequate nodal staging in early non-small cell lung cancer. Trans Lung Cancer Res 10(2):815 10.21037/tlcr-20-1024 PMC7947406 33718024

